# HSP60 and SARS-CoV-2: Les Liaisons Dangereuses

**DOI:** 10.3390/biology14091281

**Published:** 2025-09-17

**Authors:** Adelaide Carista, Melania Ionelia Gratie, Francesco Cappello, Stefano Burgio

**Affiliations:** 1 Department of Biomedicine, Neurosciences and Advanced Diagnostics (BiND), University of Palermo, 90127 Palermo, Italyfrancesco.cappello@unipa.it (F.C.); 2 Department of Medicine and Surgery, Kore University of Enna, 94100 Enna, Italy

**Keywords:** Hsp60, COVID-19, SARS-CoV-2, angiotensin-converting enzyme 2, molecular mimicry, cytokine storm

## Abstract

Heat shock protein 60 (Hsp60) plays a crucial role in cellular homeostasis and stress responses. Recent evidence highlights its involvement in COVID-19 pathophysiology, particularly in immune modulation, inflammation, and endothelial dysfunction. Extracellular Hsp60 can interact with Toll-like receptors, amplifying inflammatory responses and contributing to cytokine storm and tissue damage. Additionally, since the presence of several common epitopes with SARS-CoV-2 proteins, its role in molecular mimicry suggests a potential link also to post-infectious autoimmune disorders. Hsp60 has also been implicated in endothelial damage and thromboembolic complications observed in severe COVID-19 cases. Beyond its pathogenic roles, Hsp60 could emerge as a potential biomarker for disease severity as well as a target for therapeutic strategies aimed at modulating immune responses. Finally, the structural similarity with SARS-CoV-2 proteins raises important considerations regarding both vaccine safety and the unexpected potential for anti-tumor immunity. This review critically examines the multifaceted roles of Hsp60 in COVID-19, specifically from a morpho-functional point of view, highlighting its implications in disease progression, post-viral complications, and therapeutic opportunities.

## Simple Summary

This review examines the role of heat shock protein 60 (Hsp60) in COVID-19, highlighting its impact on immune modulation, inflammation, and endothelial dysfunction. Hsp60, a chaperone protein essential for protein folding and protection against cell stress, has been linked to COVID-19 pathogenesis through its interactions with the immune system and potential involvement in post-infectious autoimmune processes. One of the key aspects discussed is the phenomenon of molecular mimicry, wherein Hsp60 exhibits epitope similarity with a number of SARS-CoV-2 proteins, a circumstance that may potentially culminate in the induction of autoimmune responses. This mimicry has the potential to contribute to the development of autoimmune conditions, including Guillain–Barré syndrome and autoimmune thyroiditis, in patients infected with SARS-CoV-2. The document discusses Hsp60’s role in endothelial damage and thromboembolic complications, suggesting it could serve as a biomarker for disease severity and a therapeutic target. It raises concerns about vaccine safety due to Hsp60’s structural similarity to SARS-CoV-2 proteins, which may trigger autoimmune responses. The potential for Hsp60 to generate anti-tumor immunity is explored, indicating that immune responses to SARS-CoV-2 might also target tumor cells with similar epitopes. Further research is necessary to understand Hsp60’s role in disease fully and to develop effective therapeutic strategies.

## 1. Introduction

Heat shock proteins (Hsps) are a family of chaperone proteins essential for proper protein folding and cellular protection against stress [1]. Structurally characterized by a conserved GroEL-like chaperonin structure, Hsp60 forms double heptameric rings that facilitate ATP-dependent folding of nascent polypeptides. Among them, Hsp60, encoded by the *HSPD1* gene, plays a crucial role in regulating the stress response and maintaining proteostasis [2], with emerging evidence suggesting moonlighting functions in mitochondrial quality control and apoptosis regulation.

Beyond its canonical chaperoning activity, Hsp60 exerts crucial functions within mitochondria, where it ensures protein quality control and the maintenance of organelle homeostasis [3]. Importantly, Hsp60 has been shown to regulate the intrinsic apoptotic pathway, in part through its mitochondrial signal peptide, which can inhibit caspase activation and cell death in several cell types [4]. By stabilizing mitochondrial function under stress conditions, Hsp60 contributes to cell survival and adaptation. These intracellular protective effects, together with its extracellular immune-modulatory roles, underscore the dual contribution of Hsp60 to both homeostasis and pathology in viral infections such as COVID-19.

Moreover, beyond its intracellular function, Hsp60 can be released into the extracellular space through exosomal transport or passive leakage during cell necrosis, where it assumes immune system-related roles [5] as both a damage-associated molecular pattern (DAMP) and an autoantigen. This protein has been extensively studied in various pathological contexts, including inflammatory conditions like rheumatoid arthritis through Toll-like receptor (TLR)-4-mediated NF-κB activation, infective diseases such as *Helicobacter pylori* gastritis via bacterial-Hsp60 cross-reactivity, and autoimmune diseases [6,7], including type 1 diabetes mellitus, where anti-Hsp60 antibodies correlate with β-cell destruction.

The coronavirus disease 2019 (COVID-19), the SARS-CoV-2-induced pandemic, has highlighted new implications of Hsp60 in inflammatory responses and disease pathogenesis, with single-cell RNA sequencing studies revealing upregulation of HSPD1 in alveolar macrophages of severe COVID-19 patients. Its potential involvement extends to both immune mechanisms through NLRP3 inflammasome potentiation and systemic vascular damage [8,9], particularly via endothelial cell activation and complement cascade modulation. This review aims to critically analyze the multifaceted role of Hsp60 in the pathophysiology of COVID-19. We will explore the interactions of Hsp60 with the immune system, particularly its influence on dendritic cell maturation, neutrophil extracellular trap formation, and the Th17/Treg balance during viral infection.

Furthermore, the review will delve into the potential implications of Hsp60 in the development of post-infectious autoimmune processes, highlighting its role in molecular mimicry through shared epitopes with SARS-CoV-2 nucleocapsid protein and the triggering of aberrant immune responses against vascular smooth muscle cells. Additionally, Hsp60 viability as a biomarker for disease severity and progression will be evaluated through longitudinal proteomic studies, showing its elevation in fatal COVID-19 cases, as well as its promise as a therapeutic target for interventions targeting extracellular Hsp60 neutralization in acute COVID-19 infection and mitochondrial Hsp60 modulation in the so-called “long COVID syndromes”, characterized by persistent endothelial dysfunction as well as other disorders.

For this review, we first conducted a literature search covering the period from 2020 to 2025, using databases such as PubMed, Scopus, and Web of Science. The keywords employed were “Hsp60”, “COVID-19”, “SARS-CoV-2”, “molecular mimicry”, “endothelial damage”, and “cancer immunity”. Only peer-reviewed articles published in English were considered, while preprints and non-peer-reviewed reports were excluded. We then performed a second literature search covering the period from 2000 to 2025, using the same databases and applying the same inclusion and exclusion criteria, with the keywords “Hsp60”, “extra-mitochondrial functions”, “autoimmunity”, and “carcinogenesis”. The results of both searches were integrated into the writing of the paragraphs of this manuscript.

## 2. Pathogenesis of COVID-19 and the Role of Molecular Mimicry

The pathogenesis of COVID-19, caused by the SARS-CoV-2 virus, involves a multifaceted interaction between the virus and the host immune system [10]. The primary route of infection begins with the binding of the viral spike (S-) protein to the angiotensin-converting enzyme 2 (ACE-2) receptor, which is abundantly expressed on epithelial cells of the respiratory tract, as well as other tissues, including the heart, kidneys, and gastrointestinal tract [11,12]. Upon binding, the virus enters host cells via receptor-mediated endocytosis or membrane fusion, allowing the viral genome to be released and replicate within the host cell. This replication leads to viral assembly and release, which in turn activate host immune responses, including both innate and adaptive immunity.

In the context of COVID-19, molecular mimicry may contribute to the development of autoimmune responses [13,14]. Specifically, the immune system, after being triggered by the viral infection, may fail to distinguish between viral antigens and self-antigens, resulting in the production of autoantibodies or the activation of autoreactive T cells that target host tissues. This mechanism has been implicated in various post-viral autoimmune disorders observed in COVID-19 patients, including Guillain–Barré syndrome, rheumatic musculoskeletal diseases, and even the development of autoimmune thyroiditis [15,16,17]. The idea is that certain viral proteins of SARS-CoV-2, e.g., the S-protein, share epitopes with human proteins, such as those found in the nervous system or epithelial and endothelial tissues, leading the immune system to mistakenly attack these tissues.

A study examined the molecular mimicry between SARS-CoV-2 and human autoantigens involved in inflammatory polyneuropathies [18]. From the examination of the peptide sequences analyzed, it was revealed that SARS-CoV-2 shares two immunologically relevant hexapeptides (KDKKKK and EIPKEE) with human heat shock proteins 90 (Hsp90B and Hsp90B2) and 60 (Hsp60). The hexapeptide shared with Hsp90B and Hsp90B2 is among the 5 epitopes experimentally validated by SARS-CoV-2. This peptide appears to be the key to the autoimmune response against Hs90B and Hs90B2, which is a consequence of SARS-CoV-2 infection [17]. The Hsp60-recognized hexapeptide is acknowledged by lymphomonocytes from patients with demyelinating diseases of the central nervous system (CNS) [18].

The phenomenon of molecular mimicry also helps to explain some of the long-term symptoms observed in COVID-19 patients, commonly referred to as long COVID syndrome(s) or “long COVID”. In these patients, persistent immune activation—possibly triggered by molecular mimicry—may contribute to chronic inflammation and damage to various organs, leading to symptoms such as fatigue, respiratory dysfunction, and cardiovascular problems [19]. These symptoms often persist well after the acute phase of the infection has resolved, suggesting that the immune system may continue to target host tissues as a result of prolonged immune activation and autoimmunity. Furthermore, molecular mimicry may be responsible for the delayed onset of some of these complications after vaccination, as immune-mediated damage can occur weeks or even months after vaccination [20].

A study by Kovacic et al. investigated, using in silico analysis, whether peptide sequences of SARS-CoV-2 could carry out molecular mimicry mechanisms. Homologies were thus found between the viral proteins ORF1a, ORF1ab, ORF7b, and NSP3 and the human proteins PARP14, PARP9, MACROD1, and LRP2. The results of various analyses led to the conclusion that mimicking peptides can be presented to autoreactive CD8+ and CD4+ T lymphocytes, triggering an autoimmune response [20].

It has been seen that cross-reactivity with molecular mimics does not always go on to induce an immediate autoimmune response but may go on to create a ‘fertile field’. The fertile field is nothing more than the creation of a temporary immunological state that facilitates the induction of reactive T cells and can lead to the onset of autoimmune diseases [21].

Overall, while the direct viral effects of SARS-CoV-2 infection are well-documented, the potential role of molecular mimicry in the immune response highlights a more complex aspect of COVID-19 pathogenesis. Understanding the mechanisms underlying this phenomenon is crucial for developing targeted therapies that not only address the viral infection but also modulate the immune response to prevent long-term autoimmune complications and produce safer vaccines.

## 3. Hsp60, Immune Response, and Inflammation

SARS-CoV-2 infection triggers a complex immune response that, in severe cases, can lead to a cytokine storm with systemic consequences [22]. Hsp60, in addition to performing protective intracellular functions, can be released following cellular stress and activate innate and adaptive immune responses [23,24].

Recently, it has been discovered that IgM autoantibodies against several peptide epitopes of both Hsp60 and Hsp70 are present in human cord blood. From here, it was hypothesized that anti-Hsp60 and Hsp70 antibodies appear to be part of the immune system [24].

Several studies suggest that extracellular Hsp60 may act as a ligand for TLRs, particularly TLR-4, stimulating the production of pro-inflammatory cytokines, including in the lung and heart [25,26]. This activation contributes to the exacerbation of systemic inflammation observed in patients with lung diseases [25]. Furthermore, it has been hypothesized that Hsp60 may modulate macrophage activation and immune response polarization, influencing disease progression [27,28].

In this context, the mechanistic sequence can be summarized as follows: stress-induced Hsp60 release → binding to the TLR-4/CD14 complex → activation of the MyD88 adaptor → NF-κB nuclear translocation → transcription of IL-6, TNF-α, and IL-1β. This pathway, already described in inflammatory conditions of lungs, airways, and conjunctiva [29,30,31], provides a clear regulatory logic also in the case of SARS-CoV-2 infection, where excessive amplification of these cascades contributes to cytokine storm.

In the context of COVID-19, cytokine storm syndromes are discussed. In these patients, the cytokine storm causes hyperinflammation to the point of multi-organ failure. This condition is similar to secondary hemophagocytic lymphohistiocytosis, a hyperinflammatory syndrome often triggered by viral infections. In both cases, increased levels of IL-2 and IL-7, as well as other markers of inflammation, are evident. Hyperinflammation is assumed to contribute to mortality in COVID-19, evidenced by elevated ferritin and IL-6 levels in the deceased compared to survivors [28].

Another relevant aspect is the role of endogenous Hsp60 in regulating immune and inflammatory responses. Altered expression of this protein in stressed endothelial cells may contribute to tissue damage and the release of additional inflammatory mediators [32]. Emerging evidence also suggests that Hsp60 may be involved in the perpetuation of chronic inflammatory states in various anatomical districts, including the heart and lung [26,27,28,32,33,34,35], a characteristic feature of patients with persistent post-COVID-19 symptoms.

**Figure 1:**
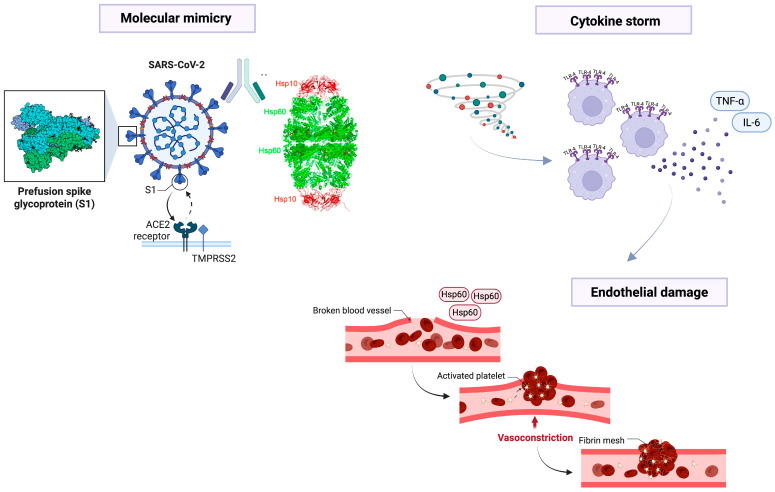
The image illustrates some mechanisms through which the virus can be pathogenic, as proposed in numerous studies and briefly summarized in this review. At the **top left**, molecular mimicry triggered by homologous epitopes between certain viral proteins and Hsp60 may induce a cytokine storm (**top right**), as well as cause direct endothelial damage (**below**), particularly if Hsp60 is localized on the plasma membrane of these cells. The former, cytokine storm, may occur when the viral spike protein interacts with the ACE-2 receptor, triggering an exaggerated immune response and the release of inflammatory cytokines such as TNF-α and IL-6. The latter, direct endothelial damage, may result from chemical (e.g., diabetes) or physical (e.g., hypertension) stress on these cells, leading to post-translational modifications of Hsp60 and its localization at the plasma membrane. This is consistent with studies by Wick et al. [39] on atherosclerosis as an autoimmune disease, induced in part by Hsp60 overexpression and its (mis)localization on endothelial cell plasma membranes. Finally, these processes lead to blood vessel damage, platelet activation, and clot formation, which can result in disseminated intravascular coagulation and, in predisposed individuals, multi-organ failure. For further information, see [51].

Hsp60 has also been involved in autoimmunity generated by molecular mimicry phenomena induced by various microorganisms [6,36,37,38,39,40] affecting different human organs and anatomical districts such as the lung, heart, nervous system, gastrointestinal tract, and many other anatomical regions. This prompted several researchers to investigate the putative involvement of Hsp60 in the pathogenesis of severe forms of COVID-19 (Figure 1), since it has been proposed that molecular mimicry is a putative pathogenetic mechanism in this disease [41,42,43]. Among them, it is interesting to highlight the studies of Jakovac [44] on hypertension, Hernandez-Cedeño et al. [45] on hyperinflammation, Mantej et al. [46] on autoantibodies, and Saha and Ahmed [47] on coagulation cascade, as these were conducted by groups independent from ours but yielded results comparable to those obtained in our studies, particularly regarding the involvement of Hsp60 in severe forms of COVID-19 [9] and post-COVID [48], as explained in more detail below.

For a more comprehensive perspective, it is also important to briefly consider the role of another important chaperone, Hsp70, in SARS-CoV-2 infection. Hsp70, differently from Hsp60, has been shown to interact directly with both the ACE2 receptor and the Spike protein’s receptor-binding domain, potentially stabilizing the S-ACE2 complex under febrile conditions and thus inhibiting viral entry into host cells (e.g., through chaperoning or masking effects) [49]. Moreover, observational studies report elevated Hsp70 levels in patients with severe COVID-19, correlated with markers of inflammation and lung injury, suggesting a role in modulating cytokine storms and ARDS [50]. These complementary functions of Hsp70 contrast with the pro-inflammatory and autoimmunity-linked roles of Hsp60, providing a more nuanced understanding of how different Hsps may exert divergent effects during COVID-19 pathogenesis.

## 4. Hsp60 and Autoimmunity During COVID-19

A growing concern is the development of autoimmune conditions in patients with SARS-CoV-2 infection. The concept of molecular mimicry has gained prominence in this context, suggesting that viral proteins may share epitopes with human proteins, including Hsp60, promoting the activation of autoimmune responses [8,9,11,12,13,14,15,16,17,18]. Particularly, in the context of infection, stress signals can lead to the overexpression of Hsp60, which, when presented on the surface of cells, may be recognized by the immune system as a target for autoreactivity, as already demonstrated in other experimental models on vascular cells [48,52,53].

It has been observed that Hsp60 can be recognized as an antigen by lymphocytes and monocytes from patients with demyelinating CNS diseases. Considering the molecular mimicry between human Hsp60 and SARS-CoV-2, the immune system may inadvertently target both the virus and human cells, potentially exacerbating CNS demyelinating processes. In 2020, a study by Zanin et al. described the case of a COVID-19 patient who was diagnosed with new demyelinating lesions. The patient, following infection with SARS-CoV-2, showed symptoms associated with neurological damage [41]. At the same time, Zhao presented a clinical case of a patient diagnosed with Guillain–Barré syndrome during SARS-CoV-2 infection [42].

Barone et al. have also identified the presence of Hsp60 in the plasma membrane of endothelial cells of the respiratory membrane of patients who died from severe COVID-19, suggesting a potential involvement of this protein in the generation of aberrant immune responses [9]. Indeed, although Hsp60 is normally present in the cytoplasm of endothelial cells, only after severe stress (e.g., shear stress, [39]) can it localize to the plasma membrane of these cells, becoming a sort of autoantigen, as it may be recognized by the immune system as a foreign molecule. This prompted us to hypothesize that autoantibodies targeting Hsp60 may lead to the formation of immune complexes that can deposit in tissues, thereby initiating inflammatory cascades. This mechanism may contribute to the tissue damage observed in various post-COVID autoimmune manifestations.

Post-COVID autoimmune manifestations include heart failure, vasculitis, and peripheral neuropathies, conditions that could be partially mediated by molecular mimicry phenomena [54,55,56]. The homology between epitopes of viral proteins and Hsp60 could initiate the activation of T cells through molecular mimicry, whereby the immune system misidentifies host proteins as foreign. Furthermore, prolonged exposure to elevated levels of extracellular Hsp60 could promote the maturation of autoreactive lymphocytes [52], which, under normal conditions, are kept in check by regulatory mechanisms. It is also possible that this process would be particularly concerning in genetically predisposed individuals, where an imbalance in immune tolerance may lead to the onset of autoimmune diseases, although this hypothesis needs further confirmation.

In addition to molecular mimicry, another potential mechanism by which Hsp60 contributes to autoimmunity is through its involvement in inflammatory cytokine release. Hsp60 can act as a DAMP that signals tissue damage and activates the immune response [53]. In infections like COVID-19, sustained elevated levels of Hsp60 could lead to a persistent inflammatory environment, which could exacerbate the immune dysregulation observed in post-viral autoimmune diseases.

However, the hypothesis of a causal link between Hsp60 and post-viral autoimmunity requires further investigation. Studies are needed to determine whether the autoimmune response to Hsp60 is a direct consequence of viral infection or whether it is exacerbated by pre-existing immune dysfunction. Moreover, the specific role of Hsp60 in the pathogenesis of autoimmune diseases following viral infections needs to be explored in more depth, particularly through longitudinal studies that can assess the timing and progression of autoantibody formation after COVID-19 infection and the severity of diseases, since a preliminary report on patients without or with mild symptoms revealed no variations [46].

## 5. Hsp60 and Endothelial Damage

Some aspects of autoimmunity related to molecular mimicry for Hsp60 are associated with the endothelium of vessels. Endothelial cells are present in the vessels of all organs. Therefore, a vascular insult to the intima layer can have potentially detrimental effects in one or more organs or throughout the human body.

SARS-CoV-2 infection has been associated with widespread endothelial dysfunction, with implications at the pulmonary, cardiac, and renal levels [57]. Hsp60 might contribute to this process through multiple mechanisms. Under oxidative stress conditions, endothelial Hsp60 expression increases, putatively facilitating inflammatory responses and immune cell recruitment [26]. Additionally, its interaction with innate immunity receptors might amplify vascular damage through the production of pro-inflammatory mediators and the promotion of endothelial permeability [55].

Recent studies have shown how SARS-CoV-2 enters cells by exploiting ACE-2 receptors, which are present on the surface of pneumocytes, thereby infecting the host and causing lung damage. A study by Varga et al. demonstrated how the virus utilizes the same highly expressed ACE-2 receptors, also present in endothelial cells, to trigger widespread endothelial dysfunction [56].

Hsp60 is also involved in the onset of endothelial damage: it can play different roles. A study conducted by Monteil et al., starting from the use of ungenerated human hair organoids, evaluated whether recombinant human soluble ACE-2 blocks the growth of SARS-CoV-2. Studies suggest that SARS-CoV-2 could go directly to infect blood vessel cells. At this point, the organoids of the infected blood vessels release the progeny of the virus. These findings suggested that inhibiting the interaction between the ACE-2 receptor and the virus could represent a potential treatment strategy for COVID-19. The experiments conducted underlined how hrsACE-2 markedly inhibited vascular organoids infected with SARS-CoV-2, demonstrating how this recombinant molecule can act, blocking the early stages of SARS-CoV-2 infection [58].

Another study, on the other hand, wants to demonstrate the involvement of endothelial cells through the vascular beds of different organs in patients with COVID-19 [56]. The action of Hsp60 can facilitate the entry of SARS-CoV-2 into endothelial cells. Hsp60, by activating the TLR-4 on endothelial cells, amplifies NF-κB signaling and the expression of adhesion molecules, such as ICAM-1, VCAM-1 [59].

Molecular mimicry induced by Hsp60 may partially explain the thromboembolic complications and microvascular alterations observed in patients with severe COVID-19 [9,42]. Understanding the role of Hsp60 in vascular damage could open new perspectives for developing therapeutic strategies aimed at reducing endothelial inflammation and the risk of cardiovascular events in COVID-19 patients.

A summary of the main aspects discussed in the last three paragraphs is provided in Table 1.

## 6. Hsp60 as a Biomarker or a Therapeutic Target

The overexpression, intracellular accumulation, and extracellular release of Hsp60 in the context of COVID-19 pathogenesis suggested its potential use as a diagnostic and prognostic biomarker. Growing evidence indicates that this chaperonin participates in both viral propagation mechanisms and host immune dysregulation, particularly through its interaction with ACE-2 receptors exploited by SARS-CoV-2 for cellular entry. Since one can expect that elevated plasma Hsp60 levels may correlate with disease severity and the risk of post-acute complications, analyzing its expression could thus provide useful information in the future for patient stratification and predicting the clinical course of the infection. Specifically, longitudinal monitoring of Hsp60 concentrations might help identify patients at risk of developing cytokine storm syndrome or persistent inflammatory states characteristic of long COVID.

However, only preliminary research has addressed this topic [46], and large-scale multicenter studies are still needed to validate cutoff values across different demographic groups while accounting for comorbidities that might influence baseline Hsp60 levels. Nevertheless, at present, the limited number of studies explains why a systematic demonstration of the biomarker value of Hsp60 is still lacking. One of the aims of this review is precisely to highlight this gap and to encourage the design of translational investigations that could move this promising field forward.

From a therapeutic standpoint, several strategies targeting Hsp60 modulation are under investigation. Inhibiting its interaction with TLRs could represent an approach to attenuate inflammation [61,62], particularly in patients showing excessive activation of the NLRP3 inflammasome pathway. Recent pharmacological developments have identified novel small-molecule inhibitors that specifically disrupt the Hsp60-TLR-4 binding interface while preserving the chaperonin’s essential protein-folding functions. To evaluate the action of Hsp60 as a biomarker, experiments were conducted to inhibit the expression of the protein. It is already known that the binding of Hsp60 to its receptor, TLR-4, is involved in the activation of the inflammatory response in microglia. A study conducted by the research group led by Xin-Lei Li et al. observed how Paraquat, a herbicide, activates inflammation in microglia by upregulating the expression of myeloid Hsp60-TLR-4, a differentiation factor, and nuclear factor-kappa B (NF-κB) [61]. By inhibiting Hsp60 expression using siRNAs or TLR-4 inhibitors, they were able to observe that the expression of TLR-4 and MyD88 and the accumulation of NF-kb in the nucleus were significantly reduced. This suggests that targeted Hsp60 suppression could synergize with existing anti-inflammatory therapies like IL-6 receptor antagonists.

## 7. Hsp60 and Vaccine Preparation

Since Hsp60 shares certain epitopes with SARS-CoV-2 [8], this raises concerns about molecular mimicry. Structural bioinformatics analyses have identified homology regions in the S-protein’s receptor-binding domain that exhibit 23% sequence similarity to conserved α-helical motifs in Hsp60’s apical domain [8]. This similarity could potentially trigger an autoimmune response in individuals, particularly those with genetic predispositions to HLA class II alleles that present these shared peptide sequences. This consideration is especially important in the development of COVID-19 vaccines, including the modern RNA-based vaccines that utilize mRNA to prompt the immune system to produce the viral S-protein.

Given that Hsp60 may play a significant role in mediating aberrant immune responses directed against host tissues, it is essential to thoroughly examine the potential interactions between the immune system and the shared epitopes present in both the SARS-CoV-2 S-protein and Hsp60, since these shared epitopes could contribute to molecular mimicry [8,43]. To minimize the risk of post-vaccination autoimmunity, a comprehensive understanding of these mechanisms is paramount. This entails investigating the structural similarities between the epitopes and how they influence T-cell and antibody responses. Advanced techniques like cryo-EM mapping of antibody–paratope interactions and single-cell TCR sequencing could identify high-risk epitope pairs requiring exclusion from vaccine constructs.

By elucidating the pathways through which Hsp60 and SARS-CoV-2 S-protein interact, researchers should develop more refined vaccine formulations [51]. This knowledge could lead to strategies that enhance vaccine safety and effectiveness, ensuring a robust immune response to the virus while simultaneously mitigating the potential for adverse autoimmune reactions through precision epitope-editing approaches or the incorporation of regulatory T-cell epitopes as immunological buffers.

Nevertheless, it should be noted that the triggering of autoimmunity differs between subjects exposed to SARS-CoV-2 without prior vaccination and those who have been vaccinated: indeed, subjects exposed to spike proteins by vaccination probably avoid the overcoming of an infective threshold (in terms of viral load) beyond which autoimmunity phenomena may become manifested with clinical signs and symptoms [54]. In other terms, vaccination predisposes the subject to react better to the infection and therefore to contain its spread to other organs other than those of the upper airways and, in turn, avoid the triggering of intravascular disseminated coagulation and, lately, multi-organ failure [42].

## 8. The Other Side of the Coin: The Anti-Tumoral Immunity

Another interesting aspect of the molecular mimicry phenomenon induced by SARS-CoV-2 is its potential role in generating anti-tumor immunity [63,64]. A study has highlighted the potential role of SARS-CoV-2 in modulating anti-tumor immunity through molecular mimicry mechanisms [63]. Proteomic analyses identified a high number of viral peptides with a very high homology to tumor-associated antigens (TAAs) across several cancer types. The results of these experiments present SARS-CoV-2 infection in a different key: while it is associated with acute complications, it could exert a long-term protective effect against tumorigenesis. The hypothesis called “natural anti-cancer vaccination” is thus presented, which defines how the immune memory generated against cross-reactive viral epitopes can monitor neoplastic cells expressing homologous TAAs [56], potentially through sustained activation of tissue-resident memory T cells (T_RM_) and enhanced interferon-γ production.

The similarity between the epitopes of Hsp60 and the SARS-CoV-2 S-protein may not only induce an autoimmune response but also stimulate an anti-tumor immune response [64], analogously to what has been proposed for Hsp60 during *Chlamydia trachomatis* infection [6]. In particular, we proposed that, for example, autoantibodies produced against Chlamydial proteins (such as GroEL), which can induce autoimmunity under certain conditions and in specific anatomical sites, could also recognize common epitopes presented by proteins localized on the tumor plasma membrane (such as Hsp60), thereby attacking and destroying these cells. In fact, it has been shown that an immune response directed against the SARS-CoV-2 virus could also lead to the recognition and destruction of tumor cells expressing similar epitopes, thus promoting an anti-tumor effect, as demonstrated in vivo [64,65]. In particular, while we had hypothesized that molecular mimicry between SARS-CoV-2 and tumoral proteins could elicit an immune reaction in which antibodies or cytotoxic cells produced against the virus cross-react with the tumor cells [64], an independent group published a report in which three patients affected by metastatic colorectal cancer after infection by SARS-CoV-2 experienced reduction of disease burden during COVID-19 course [66], inducing us to prompt clinical studies to test the hypothesis that the disease reduction in patients affected by colorectal cancer could be caused by an immune reaction against Hsps initiated by SARS-CoV-2 infection through molecular mimicry [65]. However, this phenomenon has never been demonstrated for Hsp60 until now, although we could not exclude that it would be possible.

Since this hypothesis is still under investigation, it could represent an interesting double-edged aspect of molecular mimicry, with potential positive impacts in the fight against cancer, especially in patients who may benefit from enhanced immunotherapy through epitope-masking strategies or heterologous prime-boost vaccination approaches targeting conserved tumor–viral antigen pairs.

## 9. Conclusions and Future Perspectives

Hsp60 is a putative player in COVID-19 pathophysiology, with implications ranging from immune and inflammatory responses to endothelial damage and the development of post-infectious autoimmune conditions. Table 1 summarizes all these aspects. Its role as a biomarker and potential therapeutic target represents a promising area of research. However, further studies are needed to clarify the precise mechanisms by which Hsp60 contributes to disease pathogenesis and to develop effective therapeutic strategies aimed at modulating its activity in the context of COVID-19.

For example, the structural similarity between Hsp60 and SARS-CoV-2 proteins suggests a potential role in molecular mimicry, which could lead to post-viral autoimmune responses. Hsp60 is not only implicated in endothelial damage and thromboembolic complications. Still, it could also serve as a biomarker for disease severity and as a target for therapeutic strategies aimed at modulating immune responses. Its high expression has been associated with diffuse endothelial dysfunction, with implications for the lungs, heart, and kidneys. In addition, Hsp60-induced molecular mimicry could explain the thromboembolic complications and microvascular alterations observed in patients with severe COVID-19.

In particular, as we previously proposed [51], both chemical (e.g., diabetes) and physical (e.g., hypertension) stress on endothelial cells can induce post-translational modifications of Hsp60 and its (mis)localization to the plasma membrane in these cells. In this way, Hsp60 may be recognized as an antigen by the immune system, triggering an autoimmune response against these cells (see Figure 1). This is consistent with the studies by Wick et al. [39] on atherosclerosis as an autoimmune disease, partly induced by Hsp60 overexpression and its (mis)localization on endothelial cell plasma membranes. During COVID-19, we suggest (as summarized in Figure 1) that this process may lead to blood vessel damage and platelet activation, which can result in disseminated intravascular coagulation and, in predisposed individuals, multi-organ failure [51].

Another interesting aspect is the potential role of Hsp60 in anti-cancer immunity. The similarity between the epitopes of Hsp60 and the S-protein of SARS-CoV-2 could not only induce an autoimmune response but also stimulate an anti-tumor immune response, promoting the recognition and destruction of cancer cells expressing similar epitopes. The unexpected tumor reduction observed in metastatic colorectal cancer patients during SARS-CoV-2 infection [66] supports this notion, especially considering that Hsp60 is abundantly expressed on the plasma membrane of colon cancer cells in vivo [67]. However, further studies are needed to better address this topic [64,65], possibly with a focus on novel therapeutic strategies targeting Hsp60.

Taken together, the evidence summarized in this review underscores the central and multifaceted role of Hsp60 in the pathophysiology of COVID-19. This chaperonin is not only implicated in the intracellular stress response and mitochondrial homeostasis but also emerges as an extracellular signal able to modulate innate and adaptive immunity, thereby amplifying inflammation and potentially driving cytokine storm. Its abnormal expression and mislocalization on endothelial cells may contribute to vascular damage, microthrombosis, and systemic dysfunction, which are recognized hallmarks of severe COVID-19. At the same time, the presence of molecular mimicry between Hsp60 and SARS-CoV-2 proteins provides a plausible mechanism for the emergence of post-infectious autoimmunity.

From a translational perspective, these findings support the view that Hsp60 could serve as both a biomarker of disease severity and progression, as well as a promising therapeutic target for strategies aimed at attenuating aberrant immune responses and limiting tissue damage. Moreover, the structural similarity between Hsp60 and viral proteins raises important considerations in the context of vaccine design and safety, since epitope sharing might predispose susceptible individuals to immune-mediated adverse events.

Finally, the possibility that the same mechanisms of cross-reactivity could enhance anti-tumor immunity adds a further layer of complexity, pointing to a dual role of Hsp60 in disease and health.

In conclusion, while several uncertainties remain and large-scale clinical validation is still lacking, clarifying the precise contribution of Hsp60 to COVID-19 pathogenesis and outcomes could pave the way to novel diagnostic tools and therapeutic approaches, with implications extending beyond the pandemic to other infectious, autoimmune, and neoplastic disorders.

## Figures and Tables

**Table 1:** Proposed roles of Hsp60 in COVID-19 pathogenesis. The table summarizes the main pathogenic mechanisms involving Hsp60, including its contribution to inflammation and immune response, autoimmunity, and endothelial damage, with related references.

Pathogenetic Mechanisms	Role of Hsp60 in COVID-19	Mechanistic Context	References
Inflammation and immune response	Extracellular Hsp60 acts as a ligand for TLR-4, activating NF-κB and promoting pro-inflammatory cytokine release (IL-6, TNF-α). It can modulate macrophage activation and contribute to cytokine storm.	Drives hyperinflammation and multi-organ failure in acute COVID-19; may perpetuate chronic inflammation in long-COVID.	[25,26,28,29,30,31,32,60] 11 September 2025 20:41:00
Autoimmunity	Overexpression of Hsp60 under stress leads to its presentation on the cell surface and recognition by autoreactive lymphocytes. Shared epitopes between Hsp60 and SARS-CoV-2 proteins promote molecular mimicry.	Triggers autoimmune diseases such as Guillain-Barré syndrome, CNS demyelination, vasculitis, neuropathies, and autoimmune thyroiditis.	[8,9,10,11,12,13,14,15,16,17,18,19,20,21,37,38,39,40,41,42,43,44,45,46]
Endothelial damage	Hsp60 is upregulated in endothelial cells under oxidative stress and SARS-CoV-2 infection. Its interaction with TLR-4 amplifies inflammatory cascades and adhesion molecule expression.	Contributes to endothelial dysfunction, microvascular injury, thromboembolic complications, and systemic vascular damage.	[9,26,32,55,56,57,58,59]

## Data Availability

No new data were created or analyzed in this study. Data sharing is not applicable to this article.
